# Association of Maternal Sociodemographic, Anthropometric, and Lifestyle Factors with Childhood Anthropometric Measures and Anxiety Symptoms: A Nationally Representative Cross-Sectional Study of Preschool-Aged Children in Greece

**DOI:** 10.3390/diseases13100327

**Published:** 2025-10-02

**Authors:** Exakousti-Petroula Angelakou, Athina Spyrilioti, Maria Tsiakara, Maria Vasilakaki, Constantinos Giaginis

**Affiliations:** 1 Department of Food Science and Nutrition, School of Environment, University of Aegean, 81400 Myrina, Greece; pangelakou@fns.aegean.gr; 2 Department of Statistics and Actuarial-Financial Mathematics, School of Sciences, University of the Aegean, 83200 Samos, Greece; spyrilioti.a@live.unic.ac.cy; 3 Department of Education, University of Nicosia, 2417 Nicosia, Cyprus; 4 Department of Educational Sciences and Early Childhood Education, University of Patras, 26504 Rio, Greece; 5 Department of Preschool Education Sciences and Educational Design, School of Humanities, University of the Aegean, 85100 Rhodes, Greece; psemms22029@rhodes.aegean.gr (M.T.); psemms22006@rhodes.aegean.gr (M.V.)

**Keywords:** childhood anxiety, childhood obesity, lifestyle factors, maternal factors, mental health, population well-being, preschool-aged children

## Abstract

Background/Objective: Childhood obesity and mental health disorders in preschool-aged children represent critical public health challenges with a rising global prevalence, closely linked to lifestyle behaviors and the family environment. This cross-sectional study aims to investigate the combined influence of maternal sociodemographic, socioeconomic, anthropometric characteristics, and lifestyle factors on the physical and mental health status of preschool-aged children. Methods: Validated questionnaires were administered to assess dietary habits, psychosocial parameters (depression, anxiety, stress), and sociodemographic, socioeconomic, and anthropometric variables among 200 preschool-aged children and their mothers, who served as the primary informants. Results: Maternal obesity was associated with a higher prevalence of childhood overweight/obesity (36.7% vs. 18.5% in children of non-obese mothers, *p* = 0.009). Maternal psychological factors, specifically depressive symptoms (B = 0.998, OR = 2.712, 95% CI: 1.222–6.020, *p* = 0.014) and anxiety (B = 1.676, OR = 5.346, 95% CI: 2.471–11.565, *p* < 0.001), were independently associated with an increased likelihood of child anxiety. Anthropometric measures, including waist circumference (*p* = 0.032) and hip circumference (*p* = 0.031), primarily influenced children’s physical health, whereas maternal psychological factors predominantly affected their emotional well-being. Conclusions: The findings underscore the necessity for targeted interventions focusing on enhancing maternal nutrition and mental health literacy, aiming to promote healthy dietary patterns, physical activity, and lifestyle behaviors. Such interventions are pivotal for preventing childhood obesity and fostering overall well-being at the population level.

## 1. Introduction

Obesity, defined as abnormal or excessive fat accumulation that poses a health risk [1], is now recognized as a complex, relapsing, and multifactorial neurobehavioral disease [2], resulting from intricate interactions among biological, genetic, environmental, behavioral, and social determinants [3,4,5]. In recent years, it has emerged as a major public health challenge worldwide, strongly associated with increased morbidity and premature mortality [6].

Rates of childhood overweight and obesity have also been steadily rising worldwide. In 2022, the global prevalence of overweight (including obesity) among children and adolescents aged 5–19 years reached 20%, up from 8% in 1990. In 2024, it was estimated that approximately 35 million children under the age of five were overweight [1], primarily in low- and middle-income countries [7]. In Europe, a systematic review covering 2006–2016 found a prevalence of 17.9% for overweight and 5.3% for obesity among children aged 2–7 years [8]. Southern European countries –such as Greece, Italy, Spain, and Malta– showed the highest rates of severe obesity (>4%), significantly exceeding the European average of 1–2% [9]. These geographic disparities have partially been attributed to socioeconomic factors, including low educational attainment and limited household income, both of which are associated with an increased risk of childhood obesity [10]. Greece ranks among the countries with the highest rates of childhood obesity globally [11], with a striking increase noted between preschool and early school age [12], underscoring the urgency of early preventive interventions aimed at promoting healthy behaviors.

Childhood obesity not only affects physical health but also constitutes a significant burden on children’s psychosocial and emotional development. Beyond its well-established associations with metabolic disorders, musculoskeletal problems, and increased cardiometabolic risk [3,11,13,14], a growing body of empirical evidence underscores its detrimental impact on emotional well-being and mental health, even from preschool age [5,15,16]. Specifically, children with elevated Body Mass Index (BMI) exhibit higher levels of anxiety, mood disturbances, depressive symptoms, social withdrawal, emotional dysregulation, and increased aggressive behaviors [17,18,19]. These maladaptive behaviors are not solely attributable to the physical condition per se but reflect underlying psychological difficulties associated with increased anxiety levels, emotional insecurity, and diminished self-esteem [20,21]. Moreover, exposure to stigmatizing social environments [22,23], in combination with limited opportunities for engagement in physical activities [24,25,26], exacerbates anxiety-provoking experiences, leading to social isolation and emotional destabilization. The cumulative effect of these interrelated factors increases children’s exposure to adverse life events and negatively affects their academic performance, social inclusion, and overall psychosocial well-being [24,27].

Childhood obesity is not merely the result of a positive energy balance [28], but rather represents a multifactorial phenomenon significantly influenced by family dynamics and the broader psychosocial environment [29,30]. Particular emphasis has been placed on the maternal role, as mothers constitute the primary caregivers during early childhood, critically shaping both children’s eating behaviors and emotional regulation strategies through feeding practices and the quality of interpersonal interactions [29,31,32]. This influence transcends mere dietary patterns, as maternal anthropometric status, feeding behaviors, and emotional well-being during the perinatal and early childhood periods have been associated with suboptimal outcomes in the development of healthy eating habits and emotional stability in children [33,34,35,36]. The presence of perinatal psychological distress, such as depressive symptoms and chronic anxiety, further compromises the quality of caregiving by limiting maternal emotional availability and disrupting mother-child interactions. These adverse conditions have been linked to difficulties in the development of self-regulation mechanisms, elevated emotional vulnerability, and an increased predisposition to anxiety disorders during childhood [37,38,39]. Concurrently, maternal psychological distress is associated with less supportive caregiving practices, including shorter breastfeeding duration, reduced levels of physical activity, and poorer dietary choices, wherein food is often utilized as a means of managing the child’s negative emotions [37,40]. These interrelated factors contribute to accelerated weight gain during early childhood, thereby elevating the risk of obesity and co-occurring mental health disorders, notably anxiety [41,42]. Moreover, children of mothers exhibiting persistent depressive symptomatology more frequently experience feeding difficulties, diminished emotional bonding, and lower cognitive and language skill development by the age of 3–4 years [43,44]. Similarly, maternal psychological distress, such as post-traumatic stress disorders or chronic emotional disengagement, has been correlated with compromised quality of caregiving and increased incidence of social and emotional difficulties in children, including anxiety symptoms [45,46].

These adverse factors are further exacerbated during periods of social and public health crises, such as the coronavirus disease 2019 pandemic, which imposed significant psychological and social burdens on both parents and children. The disruption of daily routines, the reduction in physical activity, and alterations in dietary habits during lockdown measures contributed to elevated levels of childhood anxiety, emotional distress, and behavioral dysregulation [47,48]. Evidence suggests that children’s adaptive capacities were substantially compromised, leading to increased susceptibility to anxiety-related responses even after the relaxation of restrictions. The transition back to normalcy was accompanied by new stressors, including intensive daily schedules, elevated academic demands, and diminished family interaction due to parental occupational obligations [49,50]. Within this context, the absence of comprehensive, multidimensional interventions at the school and community level—integrating the promotion of mental resilience, education in stress-management strategies, and the reinforcement of healthy dietary and physical activity behaviors—significantly undermines the potential for effective prevention of childhood psychological burden [51,52]. The lack of such integrative approaches fosters an environment wherein childhood anxiety tends to persist over time, interacting with other developmental and behavioral difficulties, thereby adversely affecting children’s psychosocial development and overall quality of life [53,54].

Despite increasing rates of childhood obesity, current high-quality international epidemiological data focusing on the preschool years remain limited. Many studies concentrate on older children or rely on self-reported measures, thus reducing data accuracy [8]. Furthermore, the interplay between parental obesity, mental health, and the development of obesity or psychological issues in young children remains insufficiently explored. Given that prevention is most effective during the earliest stages of life, the present study focuses on preschool-aged children in Greece and aims to investigate the relationship between maternal sociodemographic, anthropometric, and lifestyle characteristics—including psychological factors such as anxiety and depressive symptoms—and key pediatric outcomes, namely child BMI and anxiety levels. Through this, we aim to contribute to the promotion of children’s future physical and mental health.

## 2. Methods

### 2.1. Study Population

This cross-sectional study was conducted on a sample of 200 preschool-aged children (4–6.5 years) and their matched mothers, over a period of 18 months (October 2023–April 2025). Using a multistage random sampling method, participants were recruited from 13 geographical regions across Greece, including major urban centers (e.g., Athens, Thessaloniki), as well as diverse rural areas and island regions such as Crete and the North and South Aegean. This approach ensured a nationally representative sample, reflecting the country’s geographic and socio-cultural diversity.

Eligibility criteria for the included mothers having a youngest child aged between 4 and 6.5 years at the time of data collection. Participants were recruited through both public and private preschool institutions, with particular emphasis on voluntary participation and the protection of individual rights. All legal agents received comprehensive information regarding the objectives and procedures of the study and provided written informed consent in accordance with ethical standards.

Although no a priori sample size calculation was conducted, a post hoc power analysis indicated that the final sample of 200 mother–child dyads provided adequate statistical power (>80%) to detect associations of moderate effect size (Cohen’s d ≈ 0.5; OR ≈ 1.7–2.0) at the conventional significance level (α = 0.05). This sample size was deemed appropriate not only for detecting such associations but also in view of the study’s available resources and the feasible 18-month recruitment period.

Data collection and management were conducted in strict compliance with the General Data Protection Regulation [55], ensuring the privacy and confidentiality of all participants. Anonymity and data confidentiality were maintained throughout all phases of the research, from questionnaire completion to final data analysis. All procedures were conducted in accordance with the ethical standards of the institutional and national research committee and with the 1964 Helsinki Declaration and its later amendments or comparable ethical standards [56].

### 2.2. Study Design

**Figure 1:**
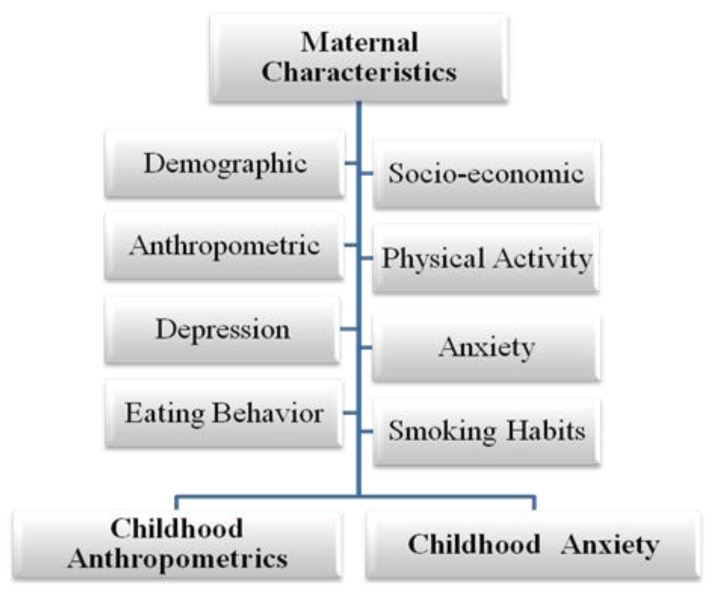
Pathways from maternal factors to anthropometric and psychological outcomes in Greek preschool-aged children.

The present study employed a cross-sectional design aiming to examine the relationship between maternal socio-demographic characteristics, anthropometric indices, and lifestyle factors with the physical and mental health status of both mothers and their preschool-aged children (Figure 1). Data was collected through structured, face-to-face interviews with the mothers, conducted primarily at home, in a familiar and comfortable setting. This approach was chosen to ensure consistency, standardization, and reliability in the data collection process [57].

During the interviews, detailed demographic (e.g., age, nationality, place of permanent residence, number, gender, and age of children, marital status) and socio-economic information (e.g., the employment status and educational level of both parents, perceived financial situation) were systematically recorded. The data collection was conducted exclusively by trained research personnel, following standardized procedures to minimize the risk of recall bias and subjective deviations [58].

Anthropometric data were collected on-site using standardized and calibrated equipment, in accordance with international protocols. For mothers, the following measurements were obtained: body weight, height, waist circumference, and hip circumference. For preschool-aged children (4–6.5 years), body weight, height, and waist circumference were measured. BMI was calculated for both mothers and children, while the Waist-to-Hip Ratio (WHR) was assessed only in mothers as an indicator of central obesity and related cardiometabolic risk [59,60]. Furthermore, mothers were asked to report their pre-pregnancy and pre-delivery weight in order to calculate pre-pregnancy and pre-delivery BMI, as well as the total gestational weight gain (GWG) during pregnancy. Weight was measured using a high-precision digital scale (accuracy ±100 g), height with a portable stadiometer (accuracy ±0.5 cm), and waist and hip circumferences with a non-elastic measuring tape. Each measurement was performed twice, and the mean value was used for analysis to minimize measurement error and ensure data accuracy.

Weight status classification for children was based on age- and sex-specific cut-off points established by the International Obesity Task Force (IOTF) [61,62]. For mothers, the World Health Organization [63] criteria were applied, categorizing individuals as underweight (BMI < 18.5 kg/m^2^), normal weight (18.5–24.9 kg/m^2^), overweight (25.0–29.9 kg/m^2^), and obese (≥30.0 kg/m^2^).

In addition to anthropometric measurements, data on lifestyle and health status were collected for both mothers and their matched children. Specifically, maternal data included dietary patterns, the presence of chronic diseases, current medication use, frequency of physical activity, and smoking habits. For children, information was gathered on any medical diagnoses, prescribed medications, nutritional issues, or developmental disorders (e.g., autism, Asperger syndrome, Rett syndrome, etc.). These variables contributed to the construction of a comprehensive family health profile, which is considered critical for the overall well-being and psychosocial development of young children [42].

To investigate the associations between maternal sociodemographic characteristics (e.g., age, nationality, place of residence, marital status, employment status, educational attainment, and household income), anthropometric indicators (BMI, WHR), and lifestyle-related factors—including psychological dimensions such as depression, anxiety/stress, dietary attitudes, physical activity, smoking status, and adherence to the Mediterranean diet—and their preschool children’s dietary habits and anxiety levels, five (5) validated and widely used diagnostic questionnaires were employed. These instruments have been standardized for the Greek population and have demonstrated strong psychometric properties, including high content validity, reliability, reproducibility, and diagnostic sensitivity, in numerous national and international epidemiological studies. No generative artificial intelligence (GenAI) tools were employed in the design, data collection, analysis, interpretation, or writing of this study.

### 2.3. Assessment of Adherence to the Mediterranean Diet

Maternal adherence to the Mediterranean diet was assessed using the validated MedDietScore questionnaire [64]. This semi-quantitative instrument evaluates the frequency of consumption across 11 key food groups, assigning a score from 0 (no consumption) to 5 (high consumption) for each component, resulting in a total score ranging from 0 to 55. Higher scores reflect greater compliance to the Mediterranean dietary pattern. The MedDietScore has demonstrated diagnostic validity through positive associations with inflammatory biomarkers, cardiometabolic risk factors, and broader indices of improved health status [65,66,67,68]. In the present study, the MedDietScore demonstrated acceptable internal consistency in our sample (Cronbach’s α = 0.760).

Preschool children’s dietary adherence was similarly evaluated using the KIDMED Index [69], a validated 16-item questionnaire designed for individuals aged 2 to 24 years. Items are scored +1 for alignment with Mediterranean diet principles and –1 for behaviors contrary to its guidelines. The resulting total score ranges from 0 to 12, with higher scores indicating better adherence to the Mediterranean dietary pattern. In our sample, the KIDMED Index showed good internal consistency (Cronbach’s α = 0.847).

### 2.4. Assessment of Depressive Symptoms

Maternal mood and potential depressive symptomatology were assessed using the Beck Depression Inventory-II (BDI-II) [70], a 21-item self-report instrument that has extensively been validated across diverse populations. The BDI-II has been translated and psychometrically standardized for the Greek population, demonstrating strong reliability and validity [71].

It evaluates depressive symptomatology across emotional (e.g., sadness, pessimism, irritability), cognitive (e.g., guilt, feelings of punishment), and somatic domains (e.g., fatigue, appetite loss, sleep disturbances, decreased libido), based on experiences over the previous two weeks. Each item is scored on a four-point Likert scale (0–3), yielding total scores from 0 to 63, with higher scores reflecting greater severity of depressive symptoms [70].

The Greek version of the BDI-II has demonstrated excellent internal consistency (Cronbach’s α = 0.93) and strong psychometric validity in both clinical and non-clinical samples [71], while international studies have confirmed its reliability and diagnostic validity (Cronbach’s α ≈ 0.90) [72,73]. In the present study, the BDI-II demonstrated high internal consistency in our sample (Cronbach’s α = 0.826), confirming its reliability for assessing depressive symptoms in Greek mothers of preschool-aged children.

### 2.5. Assessment of Anxiety Disorder

To assess anxiety profiles in both mothers and their preschool children, validated age-appropriate psychometric tools were employed. Maternal anxiety was measured using the Six-Item Short-Form of the State-Trait Anxiety Inventory (STAI-6), a concise yet psychometrically robust instrument demonstrating high internal consistency (Cronbach’s α = 0.82) and strong correlation with the full 20-item STAI (r = 0.95) [74]. In the present sample, the STAI-6 demonstrated acceptable internal consistency (Cronbach’s α = 0.764).

The STAI-6 comprises six items that assess key emotional states (calmness, relaxation, worry, happiness, distress, and tension), rated on a Likert scale based on the respondent’s present feelings. Raw scores are subsequently transformed to align with the full STAI’s scoring range (20–80), facilitating interpretability [74,75]. According to established cut-offs, scores between 34 and 37 reflect normal anxiety levels, whereas scores ≥38 suggest heightened anxiety and potential clinical relevance [76].

Preschool children’s anxiety was assessed using the State-Trait Anxiety Inventory for Children (STAI-CH) [77], a widely employed psychometric tool that has been culturally adapted and validated for Greek populations [78]. The instrument comprises 40 items, organized into two subscales: (a) State Anxiety (A-State), which captures the individual’s current, transient emotional state, and (b) Trait Anxiety (A-Trait), which assesses the individual’s stable emotional predisposition (i.e., dispositional or enduring anxiety). Responses are provided on a three-point Likert-type scale, reflecting the intensity of experienced emotions. Each subscale yields a score ranging from 20 to 80, while the overall anxiety index ranges from 40 to 160, with higher scores indicating increased levels of anxiety [77].

Given the limited cognitive and linguistic maturity of preschool-aged children, the STAI-CH was completed via maternal proxy-reporting, in line with internationally endorsed methodological protocols recommending parental assessments for this age group [79,80]. Proxy reports have been shown to validly reflect internalizing symptoms in young children, particularly when administered under the supervision of trained research personnel [81]. In our sample, the STAI-CH demonstrated good internal consistency (Cronbach’s α = 0.858 for the overall scale). Mothers received structured guidance from experienced staff to ensure accurate comprehension of the items, while avoiding bias in their responses. This methodological approach is further supported by literature demonstrating high concordance between parent-reported data and children’s internal emotional states, especially in early developmental stages [82].

All questionnaires were administered in a structured sequence under the guidance of trained research personnel, who provided standardized instructions, clarifications, and practical examples to minimize potential misunderstandings and optimize response accuracy. Furthermore, in line with ethical research practice and to enhance participant engagement, mothers received individualized feedback based on their questionnaire results, along with tailored advice on nutrition and mental health. This approach aimed to strengthen rapport with participants and promote trust in the data collection process [83].

### 2.6. Statistical Analysis

Descriptive statistics were used to summarize the main characteristics of the study variables. The normality of continuous variables—including psychological scores (depression and anxiety) and indices of adherence to the Mediterranean diet—was assessed using the Kolmogorov–Smirnov test. For normally distributed variables, group comparisons were performed using Student’s t-test, whereas the Kruskal–Wallis and Mann–Whitney U tests were applied for variables that did not follow a normal distribution. To investigate the independent associations between adherence to the Mediterranean diet and psychological outcomes with key demographic, socio-economic, and anthropometric factors, multivariable binary logistic regression analyses were conducted, adjusting for potential confounding variables. Additionally, multivariable logistic regression analyses were performed to examine the independent associations between maternal characteristics and the presence of anxiety symptoms in children, controlling for relevant confounders. Although subgroup or interaction analyses were not performed in this study, the main analyses were adjusted for key potential confounders as described above. Sensitivity analyses were not conducted; however, data completeness was verified, and no missing data were present. The multistage sampling design ensured a nationally representative sample of preschool-aged children and their mothers across Greece; however, the hierarchical structure of the sample was not explicitly incorporated into the statistical models. Statistical significance was set at *p* < 0.05. All statistical analyses were performed using Statistica software, version 10.0 (Informer Technologies, Hamburg, Germany).

**Figure 2:**
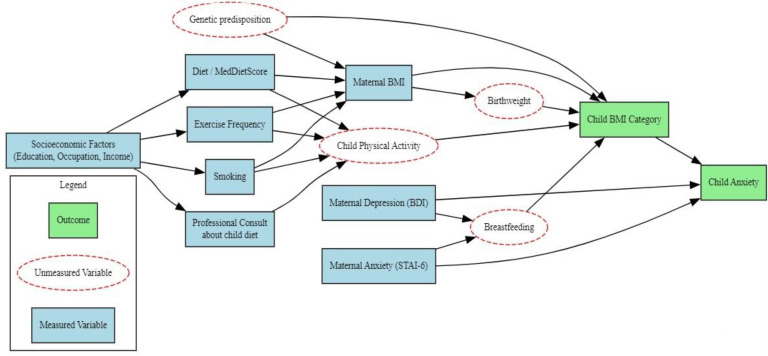
Directed Acyclic Graph (DAG) for confounding control and variable selection. The DAG illustrates the hypothesized relationships among variables included in the multivariable model. Outcomes are represented in green boxes, measured maternal covariates in blue boxes, and key unmeasured potential confounders in red dashed boxes. This graphical representation guided the identification of minimal sufficient adjustment sets to control for confounding while avoiding adjustment for mediators or colliders.

To inform covariate selection for the multivariable model, a Directed Acyclic Graph (DAG) was constructed in accordance with established literature [84]. The DAG incorporated all measured maternal variables, including BMI, anthropometric parameters, socioeconomic indicators, physical activity, smoking status, dietary and psychological measures, as well as professional guidance regarding the child’s diet. The dependent variables comprised child BMI categories and the presence of anxiety. Furthermore, key but unmeasured potential confounders, such as genetic predisposition, birthweight, breastfeeding, and child physical activity, were explicitly represented. This graphical approach facilitated the identification of minimal sufficient adjustment sets necessary to control for confounding while avoiding adjustment for mediators or colliders [84]. Guided by the DAG structure, the final set of variables included in the multivariable model was selected to minimize bias and ensure robust estimates of causal effects [84]. Accordingly, the model specification was fully justified based on the DAG, which is presented in Figure 2.

## 3. Results

### 3.1. Descriptive Statistics of the Study Population

The present study included 200 preschool-aged children living in Greece and their matched mothers, who served as the primary source of data through structured interviews and standardized questionnaires. Regarding place of residence, the vast majority of mothers (85.5%) reported living in rural areas, while 14.5% resided in urban centers. The overwhelming majority of participants were of Greek nationality (95.5%), with a small minority (4.5%) being non-Greek nationals. In terms of employment status, 81.4% of mothers were employed at the time of data collection, whereas 18.6% reported being unemployed. The age distribution revealed that most mothers (61%) were between 35 and 42 years of age, followed by 23% aged 23–34 years, and 16% over 43 years, indicating a relatively mature maternal profile. With regard to educational attainment, the majority of mothers (67.5%) had completed higher or tertiary education, while 30% had completed secondary education, and only 2.5% reported lower educational levels. As for marital status, most participants were married (94%), while 2.5% were single, and 3.5% divorced. In terms of family composition, 79.5% of mothers reported having one or two children, 17% had three to four children, and 3.5% had five or more, suggesting predominantly small to medium-sized family structures. Self-reported economic status was rated as moderate by 52.5% of the respondents, low by 41%, and high by only 6.5%.

Regarding the demographic characteristics of the preschool-aged children, girls constituted the majority (55.5%), while boys accounted for 44.5%. The age distribution revealed that the largest proportion of children belonged to the 4–5-year age group (61.5%), followed by the 5–6-year group (28.5%), and the 6–6.5-year group (10%). This distribution mirrors the typical age composition of the early childhood population, with a clear concentration in the 4–5-year range, which often corresponds to the core years of preschool attendance.

### 3.2. Anthropometric Characteristics, Health Indicators, and Lifestyle Factors of the Study Population

#### 3.2.1. Maternal Health Indicators and Anthropometric Characteristics

The majority of participating mothers reported being non-smokers (79.5%) and having no diagnosed medical conditions at the time of data collection (80.5%). Nonetheless, 16.5% of the mothers reported regular use of prescribed medication, primarily to manage chronic conditions such as hypothyroidism, Hashimoto’s thyroiditis, beta-thalassemia trait, and autoimmune psoriatic arthritis.

Regarding body composition, most mothers were classified within the normal BMI range (57.5%), while 27.5% were overweight and 15% were obese. Importantly, substantial changes in body weight were observed during pregnancy. Prior to conception, 79% of mothers fell within the normal BMI category; however, this proportion declined sharply to 29% by the time of delivery. Correspondingly, the prevalence of overweight and obesity increased to 44.5% and 26.5%, respectively. These notable shifts underscore the potential impact of GWG, especially when adequate guidance and monitoring are lacking.

Concerning body circumferences, 42.5% of participants reported a waist circumference between 76 and 90 cm, while 55.5% had a hip circumference ranging from 96 to 110 cm. The WHR, a key indicator of fat distribution and associated health risk, was predominantly within the physiologically normal range for females (0.76–0.85) in 55.5% of the sample. In terms of stature, the height distribution was relatively balanced, with the majority of mothers (55.5%) falling within the study-defined average height category.

In summary, the maternal health and anthropometric indicators portray an overall favorable profile. Nevertheless, a significant subset of participants displays weight-related risk factors that may benefit from targeted preventive or corrective interventions aimed at improving long-term health outcomes.

#### 3.2.2. Children’s Health Indicators and Anthropometric Characteristics

The majority of children in the sample were reported to have no diagnosed health issues (95.5%) and were not receiving regular pharmacological treatment (98.5%). Within the subset of children with reported difficulties (4.5%), 2% had developmental or neurological disorders (e.g., autism, ADHD, speech delay), while 2.5% presented physical or organic conditions (e.g., allergies, asthma, eczema, iron-deficiency anemia, bronchitis).

Regarding anthropometric measures, most children weighed between 17.5 and 24 kg (60.5%) and were 105.5 to 118 cm tall (50.5%). Waist circumference predominantly ranged from 50.5 to 57 cm (57%). Weight classification, based on age- and sex-adjusted thresholds from the International Obesity Task Force (IOTF), indicated that 84.5% of children were of normal weight, with 11% classified as overweight and 4.5% as obese.

Overall, the above findings suggest a generally positive health and nutritional profile among the children. However, the presence of overweight and obesity, though limited, underscores the need for ongoing monitoring and early intervention focusing on childhood nutrition and physical activity.

#### 3.2.3. Lifestyle Factors: Nutrition, Mental Well-Being, and Daily Routines of Mothers

To better understand the lifestyle of the participating mothers, key health-related behaviors were examined, including dietary habits, physical activity levels, and mental well-being. Regarding dietary habits, the vast majority of mothers (95.5%) reported not following any specific diet. However, findings from the MedDietScore revealed that 73% of mothers demonstrated high adherence to the Mediterranean dietary pattern, indicating the adoption of healthy eating behaviors.

In terms of physical activity, 37.5% of mothers reported complete absence of physical exercise, 33.5% reported low engagement, and only 29% reported regular involvement in some form of physical activity.

With respect to mental health, 78% of the participants reported few or no depressive symptoms, indicating a generally positive psychological state, while 22% exhibited signs of depressive symptomatology and, consequently, poorer psychological well-being. Anxiety levels were nearly evenly distributed, with 50.5% of mothers experiencing high anxiety levels and 49.5% reporting normal levels.

Overall, the findings portray a multifaceted lifestyle profile. While most mothers appear to adopt healthy dietary practices, limited physical activity and considerable psychological burden underscore the need for targeted prevention and health promotion efforts, particularly among women raising preschool-aged children.

#### 3.2.4. Children’s Dietary Behaviors and Anxiety Levels

To assess the emotional and psychological status of the children, their dietary habits as well as their anxiety levels were examined. Regarding nutrition, the majority of mothers (54.5%) reported that their children demonstrated a moderate level of adherence to the Mediterranean Diet, as assessed by the KIDMED Score, indicating a moderate dietary quality. Additionally, 40% of mothers reported high adherence to the Mediterranean dietary pattern, while only 5.5% reported low adherence. Notably, only 12.5% of mothers had sought guidance from a healthcare professional regarding their children’s nutritional needs, whereas the majority (87.5%) had not received such support, an observation that may be linked to the moderate dietary quality observed.

Concerning anxiety levels, 68.5% of the children were categorized as having low anxiety, 26.5% presented moderate anxiety levels, and only 5% belonged to the high-anxiety group. These findings suggest that the majority of participating children exhibit satisfactory dietary habits and low levels of anxiety, both of which may contribute positively to their emotional and psychological well-being.

### 3.3. Comparative Analysis of Demographic, Anthropometric, Psychological, and Lifestyle Characteristics of Mothers in Relation to Children’s BMI Categories

To investigate the relationship between maternal characteristics and children’s BMI, which was categorized into three groups (underweight, normal weight, and overweight/obese), a range of independent variables were examined. These included demographic, socioeconomic, and anthropometric characteristics of mothers, as well as psychological factors and lifestyle parameters. All variables were evaluated using *p*-values to identify statistically significant associations and determine the level of significance (Table 1).

Regarding demographic characteristics, no statistically significant association was observed between maternal age and children’s BMI (*p* = 0.486), as the different age groups of the children showed similar distribution across the three BMI categories. Non-parametric tests were applied due to the nature of the data and violations of normality assumptions. The Kruskal–Wallis analysis (H = 0.605, *p* = 0.739) revealed no statistically significant differences between maternal age and the three BMI categories of children (underweight, normal weight, overweight/obese). Although the mean ranks were slightly higher for mothers of underweight children (Mean Rank = 107.08), this difference did not reach statistical significance.

Similarly, no significant associations were found for permanent residence (*p* = 0.947), nationality (*p* = 0.320), number of children (*p* = 0.435), physical activity frequency (*p* = 0.409), smoking habits (*p* = 0.439), presence of illness (*p* = 0.088), use of medication (*p* = 0.073), as well as other variables such as maternal education level (*p* = 0.654), occupation (*p* = 0.507), dietary habits (*p* = 0.607), mood status (*p* = 0.856), and anxiety levels (*p* = 0.525).

In contrast, certain maternal anthropometric characteristics demonstrated statistically significant or marginally significant associations with children’s BMI. Specifically, the WHR showed marginal significance (*p* = 0.061), with higher WHR values being associated with increased rates of childhood overweight or obesity. A similar marginal trend was observed for maternal pre-pregnancy weight (*p* = 0.052), suggesting a possible effect of pre-gestational maternal weight on the child’s future body weight. On the other hand, the Kruskal–Wallis analysis of GWG did not reveal statistically significant differences between the BMI categories of children (H = 3.494, *p* = 0.174), despite a trend of higher mean rank values among mothers of overweight/obese children (Mean Rank = 114.82). Additionally, normality testing was performed for the variables “Maternal age” and “Maternal GWG” using the Kolmogorov–Smirnov test, which indicated that neither variable followed a normal distribution, as the significance level was below 5%.

A statistically significant association was found for current maternal body weight (*p* = 0.013), with the prevalence of childhood overweight/obesity increasing proportionally with maternal weight. Specifically, mothers weighing more than 75 kg had overweight/obese children at a rate of 26.9%, compared to 14.5% in mothers weighing 61–75 kg, and only 8.3% in mothers weighing 44–60 kg. A similar significant pattern was observed for maternal BMI (*p* = 0.009). Among mothers with normal BMI, 9.6% of children were overweight/obese, increasing to 16.4% among overweight mothers and reaching 36.7% among obese mothers, suggesting a strong intergenerational and/or environmental influence on child body weight.

Furthermore, maternal waist circumference was significantly associated with child BMI (*p* = 0.032). Mothers with waist circumference between 60–75 cm had children with increased weight at a rate of 8.8%, which rose to 12.9% for 76–90 cm, and 25.9% for waist circumference over 91 cm. Likewise, maternal hip circumference (*p* = 0.031) was associated with children’s BMI: mothers with a hip circumference of 70–95 cm had overweight/obese children at a rate of 12.5%, 11.7% for 96–110 cm, and a significant increase to 33.3% for those over 111 cm.

Marital status also emerged as a significant factor (*p* = 0.017). Specifically, among married mothers, the percentage of children with excessive weight (overweight and obesity) was 14.4%, while in divorced mothers this percentage increased to 57.1%, indicating a possible influence of psychosocial and family factors in the development of childhood obesity.

### 3.4. Association of Maternal Socio-Demographic, Anthropometric, Psychological, and Lifestyle Characteristics with Children’s Anxiety Levels

#### 3.4.1. Association Between Maternal Factors and Children’s Anxiety

The analysis of the relationship between maternal socio-demographic and anthropometric characteristics, as well as lifestyle factors—including dietary habits, general health, and mental health—and the presence of anxiety in their children revealed that none of the socio-demographic or physical variables showed statistically significant differences between the two groups (children with and without anxiety), except for two psychological parameters (Table 2). Specifically, maternal age did not differ significantly between groups (mean age approximately 38 years in both groups, *p* = 0.213). Similarly, place of residence, predominantly rural (median = 2 in both groups), was not associated with the presence of anxiety in children (*p* = 0.616). Furthermore, factors such as nationality (predominantly Greek), marital status, maternal occupation, economic status, and educational level showed no significant differences between groups (all *p* > 0.05), despite their theoretical association with child psychological well-being.

Maternal anthropometric parameters, including weight, height, BMI, waist and hip circumferences, and WHR, demonstrated comparable median values between groups without statistically significant differences. For example, BMI ranged overall around 24.87, with medians of 23.98 and 23.77 for the anxiety and non-anxiety groups, respectively (*p* = 0.857). GWG was numerically higher in the anxiety group (median = 13) compared to the non-anxiety group (median = 10), though this difference was not statistically significant (*p* = 0.074), potentially indicating a trend warranting further investigation in a larger sample.

Regarding lifestyle indicators, neither weekly frequency of physical exercise (*p* = 0.937) nor smoking habits (*p* = 0.333) differed significantly between groups. Similar non-significant associations were observed for the presence of chronic disease, medication use, diet type, adherence to the Mediterranean Diet (MedDietScore), and family status, despite their theoretical links to overall well-being and family environment.

In contrast, two maternal psychological parameters demonstrated a statistically robust association with the presence of anxiety in the child. Specifically, maternal depressive symptomatology, as assessed by the BDI-II, differed significantly between the two groups (*p* < 0.001), with mothers of anxious children exhibiting higher levels of depressive symptoms. Similarly, maternal anxiety, as measured by the STAI-6, was significantly elevated among mothers of children with anxiety (*p* < 0.001).

Finally, seeking professional counseling or psychological support did not differ between groups (*p* = 1.000), suggesting a gap in access to or activation of supportive mechanisms irrespective of maternal or child psychosocial burden.

In summary, while most maternal socio-demographic, anthropometric, and behavioral factors were not associated with anxiety manifestation in children, maternal psychological variables—particularly depressive symptoms and anxiety levels—emerged as significant contributors. These results underscore the importance of focusing on psychological dimensions within preventive and supportive interventions aimed at improving children’s mental health.

#### 3.4.2. Multivariable Logistic Regression Analysis of Maternal Predictors of Anxiety Absence in Preschool-Aged Children

Multivariable logistic regression was conducted to evaluate the effect of selected maternal parameters—both anthropometric and psychological—on the likelihood of absence of anxiety in the child, using the presence of anxiety as the reference category. Results are presented with regression coefficients (B), standard errors, odds ratios (OR), 95% confidence intervals (CI), and *p*-values (Table 3).

The constant was negative (B = −4.017, *p* < 0.001), indicating a low baseline probability of absence of anxiety when all predictors equaled zero. However, the absolute interpretation of this finding depended on the measurement scale and centering of the variables. Among the predictors, maternal depression (B = 0.998, *p* = 0.014, OR = 2.712, 95% CI: 1.222–6.020) and—more prominently—maternal anxiety (B = 1.676, *p* < 0.001, OR = 5.346, 95% CI: 2.471–11.565) emerged as independent predictors of child anxiety status. Specifically, each one-unit increase in maternal depression score was associated with a 2.7-fold higher likelihood of the child being classified as anxious, while each one-unit increase in maternal anxiety score was associated with more than a fivefold higher likelihood, holding all other variables constant. Both associations were statistically robust, as the corresponding confidence intervals did not include unity.

In contrast, pre-pregnancy maternal weight (B = −0.376, *p* = 0.189, OR = 0.687, 95% CI: 0.392–1.202), maternal height (B = 0.225, *p* = 0.442, OR = 1.253, 95% CI: 0.705–2.226), and GWG (B = −0.041, *p* = 0.145, OR = 0.960, 95% CI: 0.908–1.014) were not significantly associated with child anxiety status, as their confidence intervals encompassed unity and Wald tests failed to reject the null hypothesis.

## 4. Discussion

In the context of growing global concern regarding childhood obesity and mental health issues—most notably anxiety—this cross-sectional descriptive study explored the associations between maternal characteristics and key indicators of physical and mental health in preschool-aged children. Specifically, the study examined the relationships between maternal sociodemographic and anthropometric attributes, as well as lifestyle factors (including dietary habits, general health status, and maternal psychological well-being), with children’s BMI and levels of anxiety.

Despite the increasing scientific focus on childhood obesity and mental health, the preschool period remains underrepresented in international research. Existing studies frequently exhibit methodological limitations, such as geographical confinement, reliance on self-reported data, or the isolated examination of single parameters. Consequently, there is a pressing need for a holistic, multifactorial approach that addresses the complex, multilayered influence of the maternal environment on child health outcomes.

The findings of the present study revealed selective yet statistically significant associations between maternal factors and key indicators of physical and mental health in preschool-aged children. Specifically, elevated maternal BMI, increased waist and hip circumference, higher pre-pregnancy weight, and an elevated WHR were positively correlated with the presence of overweight or obesity in children. Additionally, maternal marital status appeared to influence child BMI, with higher rates of overweight observed among children of divorced mothers.

At the psychosocial level, maternal mental health—particularly depressive symptoms and elevated anxiety levels—was associated with an increased likelihood of emotional difficulties in children. Specifically, offspring of mothers experiencing high psychological distress appeared to be at greater risk of developing anxiety-related symptoms. These findings underscore the pivotal role of maternal psychological well-being in shaping children’s emotional development and highlight the importance of targeted interventions aimed at improving maternal mental health.

The findings of the present study align with prior research investigating the association between maternal characteristics and both physical and mental health outcomes in preschool and school-aged children. The positive correlation between maternal body weight—current, pre-pregnancy, and gestational—and the increased risk of childhood overweight has been well-documented in numerous international studies, which consistently identify elevated maternal BMI as a significant predictor of childhood obesity, potentially through intergenerational metabolic programming mechanisms [85,86]. Prospective data further substantiate that both pre-pregnancy BMI and excessive GWG are associated with accelerated weight gain trajectories and elevated BMI as early as preschool age, with the impact potentially intensifying during preadolescence due to the stabilization of obesity patterns [87,88]. Moreover, population-based studies from Asia, Europe, and Greece (GRECO study) have underscored maternal obesity as a critical determinant in the early onset and establishment of childhood obesity [89,90,91,92]. These data highlight the imperative for the development of preventive interventions focusing on prenatal care and maternal support as strategic pillars in curbing childhood obesity at a population level.

Furthermore, the observed association between elevated maternal adiposity indicators (waist circumference, hip circumference, and WHR) and the prevalence of overweight or obesity in preschool-aged children is corroborated by existing international literature. Empirical evidence underscores central obesity markers as independent predictors of increased birth weight and early-life obesity indicators, irrespective of maternal BMI, socioeconomic status, or gestational age [93,94,95]. Additionally, longitudinal and population-based studies have consistently demonstrated a positive relationship between maternal waist circumference and increased adipose tissue accumulation in offspring, from infancy through early childhood (up to approximately 8 years of age) [96,97,98,99]. These findings reinforce the notion that maternal fat distribution constitutes a critical biological determinant in the early metabolic programming of the child.

Moreover, the present study identified an elevated risk of childhood overweight among offspring of divorced mothers compared to those of married mothers, a finding substantiated by prior research. The transition from a biparental to a single-parent family structure has been associated with increased likelihood of both general and abdominal obesity, independent of socioeconomic and cultural determinants [100,101]. Specifically, studies conducted in Norwegian populations have reported higher prevalence rates of childhood obesity among children of divorced parents, with a more pronounced effect observed in boys [102]. Prolonged residence in single-parent households or the absence of paternal involvement has also been linked to elevated BMI trajectories starting as early as toddlerhood [103,104]. In Greece, findings from the GENDAI study highlighted the adverse impact of family dissolution on both children’s physical health and dietary behaviors, often shaped by emotional determinants [105]. Collectively, family instability emerges as a significant psychosocial and environmental risk factor for the development of childhood obesity.

Anxiety constitutes one of the most prevalent psychological disorders in childhood, with an increasing incidence manifesting from the earliest developmental stages. The findings of the present study are consistent with the existing literature, which highlights the frequent and persistent nature of anxiety symptoms in the preschool years. Systematic reviews indicate that approximately 17.6% of preschool-aged children exhibit mental health difficulties, with 25–67% of these symptoms persisting beyond a three-year period [106].

Our study revealed a significant association between maternal depressive symptomatology and elevated levels of anxiety in children, corroborating previous research findings. Maternal depression has been consistently linked to heightened psychopathology in offspring, encompassing both internalizing symptoms (anxiety, depression) and externalizing behaviors (aggression), as well as generalized emotional dysregulation [107]. In early childhood, exposure to a depressive familial environment appears to disrupt the development of emotional self-regulation, leading to increased vulnerability not only to transient anxiety responses but also to the formation of stable anxious temperaments [108,109].

Similarly, maternal anxiety emerged as a strong independent predictor of child anxiety. Higher maternal anxiety were associated with significantly increased odds of the child being classified as anxious, consistent with the majority of international literature demonstrating positive associations between maternal anxiety and the manifestation of anxiety symptoms in children [107,110,111]. These findings reinforce the critical role of maternal psychological well-being in shaping early emotional development.

Taken together, these observations underscore maternal psychological health—particularly depressive symptoms and anxiety—as key determinants of children’s emotional well-being. These results are consistent with and extend existing theoretical models of intergenerational transmission of psychological distress, highlighting the importance of integrating maternal mental health interventions into preventive strategies aimed at reducing emotional disorders during early childhood.

The present study exhibits several notable strengths that enhance its scientific rigor and the validity of its findings. The geographically representative sampling of preschool children and their mothers from both urban and rural areas of Greece increases the generalizability of the results. Furthermore, the multifactorial approach, integrating sociodemographic and anthropometric indicators alongside lifestyle factors, enabled the exploration of complex associations. An additional advantage was the employment of validated instruments with established psychometric reliability. Objective measurement collection and standardized interviews conducted by trained personnel further bolstered data reliability. A significant contribution of this research is the identification of the association between maternal WHR and indicators of childhood anxiety. Moreover, the provision of individualized feedback to mothers facilitated engagement and enhanced trust during data collection.

Despite these strengths, several limitations should be considered when interpreting the results. The cross-sectional design precludes causal inferences, as the observed relationships represent associations rather than causative links. The use of self-report instruments, notwithstanding their psychometric validation, may introduce recall bias and social desirability effects, particularly regarding parental assessments of child anxiety. The complexity of multifactorial models also carries the risk of uncontrolled confounding or mediating variables. The exclusive focus on maternal data, without inclusion of paternal or other caregiver information, limits the comprehensiveness of the familial context portrayed. Moreover, the sample consisted of 200 children and their mothers, and although participants were drawn from 13 regions, the distribution was uneven, potentially affecting representativeness and generalizability. Additionally, the personal acquaintance of the researchers with some participants may have contributed to more favorable responses, despite anonymity, increasing the risk of social desirability bias. Finally, future research could expand to larger and more diverse populations and implement longitudinal designs to enhance the practical applications of the findings for interventions targeting child anxiety and maternal health.

## 5. Conclusions

This study is among the few cross-sectional investigations to comprehensively explore the combined influence of maternal demographic, socio-economic, anthropometric, and lifestyle-related factors—including dietary behavior, general health status, and psychological well-being—on the physical and mental health outcomes of preschool-aged children. The findings highlight a significant association between maternal obesity and an increased risk of overweight and obesity in children, underscoring the relevance of early-life metabolic programming within the family context. Additionally, maternal depressive symptoms were significantly associated with higher anxiety levels in preschool-aged children, emphasizing the intergenerational impact of maternal mental health on children’s psychosocial development. While anthropometric indicators such as BMI, WHR, and pre-pregnancy weight were mainly linked to physical outcomes, psychological factors were more closely related to children’s emotional well-being. These results support the need for integrated, family-centered interventions targeting both maternal physical and mental health as a means to promote holistic child development. School-based health education programs, including parental and educator training in nutrition, mental health, and lifestyle quality, may serve as effective primary prevention strategies for childhood obesity and anxiety. Finally, well-designed longitudinal population-based studies are needed to clarify the causal pathways linking maternal characteristics, such as WHR, to child health outcomes in early life.

## Figures and Tables

**Table 1:** Analysis of the correlation between maternal characteristics and children’s BMI.

Independent Maternal Variables		N	Category A: BMI—Underweight Children	Category B: BMI—Children with Normal Weight	Category C: BMI—Overweight & Obese Children	*p*
**Age**	23–34 years old	46	4 (8.7%)	33 (71.7%)	9 (19.6%)	0.486
	35–42 years old	122	24 (19.7%)	80 (65.6%)	18 (14.8%)	
	43+ years old	32	5 (15.6%)	23 (71.9%)	4 (12.5%)	
**Permanent residence**	Urban	29	5 (17.2%)	19 (65.5%)	5 (17.2%)	0.947
	Rural	171	28 (16.4%)	117 (68.4%)	26 (15.2%)	
**Nationality**	Greek	191	33 (17.3%)	128 (67%)	30 (15.7%)	0.320
	Non-Greek	9	0 (0%)	8 (88.9%)	1 (11.1%)	
**Number of children**	1–2 children	159	25 (15.7%)	109 (68.6%)	25 (15.7%)	0.435
	3–4 children	34	5 (14.7%)	24 (70.6%)	5 (14.7%)	
	5+ children	7	3 (42.9%)	3 (42.9%)	1 (14.3%)	
**Weight**	44–60 kg	72	9 (12.5%)	57 (79.2%)	6 (8.3%)	0.013
	61–75 kg	76	18 (23.7%)	47 (61.8%)	11 (14.5%)	
	75+ kg	52	6 (11.5%)	32 (61.5%)	14 (26.9%)	
**Height**	1.50–1.60 m	60	10 (16.7%)	41 (68.3%)	9 (15%)	0.133
	1.61–1.70 m	111	18 (16.2%)	71 (64%)	22 (19.8%)	
	1.71+ m	29	5 (17.2%)	24 (82.8%)	0 (0%)	
**BMI**	Normal weight	115	21 (18.3%)	83 (72.2%)	11 (9.6%)	0.009
	Overweight	55	9 (16.4%)	37 (67.3%)	9 (16.4%)	
	Obesity	30	3 (10%)	16 (53.3%)	11 (36.7%)	
**Waist circumference**	60–75 cm	57	6 (10.5%)	46 (80.7%)	5 (8.8%)	0.032
	76–90 cm	85	18 (21.2%)	56 (65.9%)	11 (12.9%)	
	91+ cm	58	9 (15.5%)	34 (58.6%)	15 (25.9%)	
**Hip circumference**	70–95 cm	56	8 (14.3%)	41 (73.2%)	7 (12.5%)	0.031
	96–110 cm	111	19 (17.1%)	79 (71.2%)	13 (11.7%)	
	111+ cm	33	6 (18.2%)	16 (48.5%)	11 (33.3%)	
**WHR**	≥0.75	35	5 (14.3%)	28 (80%)	2 (5.7%)	0.061
	0.76–0.85	111	22 (19.8%)	74 (66.7%)	15 (13.5%)	
	0.86+	54	6 (11.1%)	34 (63%)	14 (25.9%)	
**Pre-pregnancy weight**	44–55 kg	60	8 (13.3%)	46 (76.7%)	6 (10%)	0.052
	56–70 kg	104	23 (22.1%)	65 (62.5%)	16 (15.4%)	
	71+ kg	36	2 (5.6%)	25 (69.4%)	9 (25%)	
**Weight before delivery**	50–65 kg	47	4 (8.5%)	38 (80.9%)	5 (10.6%)	0.176
	66–80 kg	96	20 (20.8%)	62 (64.6%)	14 (14.6%)	
	81+ kg	57	9 (15.8%)	36 (63.2%)	12 (21.1%)	
**Frequency of exercise/week**	None	75	13 (17.3%)	48 (64%)	14 (18.7%)	0.409
	A little	67	12 (17.9%)	43 (64.2%)	12 (17.9%)	
	Often	58	8 (13.8%)	45 (77.6%)	5 (8.6%)	
**Smoking**	Yes	41	6 (14.6%)	31 (75.6%)	4 (9.8%)	0.439
	No	159	27 (17%)	105 (66%)	27 (17%)	
**Illness**	Yes	39	2 (5.1%)	29 (74.4%)	8 (20.5%)	0.088
	No	161	31 (19.3%)	107 (66.5%)	23 (14.3%)	
**Medication use**	Yes	33	3 (9.1%)	22 (66.7%)	8 (24.2%)	0.073
	No	167	30 (18%)	114 (68.3%)	23 (13.8%)	
**Diet (type)**	Non-specific diet	191	33 (17.3%)	127 (66.5%)	31 (16.2%)	0.109
	Special diet	9	0 (0%)	9 (100%)	0 (0%)	
**Marital status**	Single	5	0 (0%)	5 (100%)	0 (0%)	0.017
	Married	188	32 (17%)	129 (68.6%)	27 (14.4%)	
	Divorced	7	1 (14.3%)	2 (28.6%)	4 (57.1%)	
**Economic status**	Low	82	15 (18.3%)	53 (64.6%)	14 (17.1%)	0.837
	Medium	105	15 (14.3%)	75 (71.4%)	15 (14.3%)	
	High	13	3 (23.1%)	8 (61.5%)	2 (15.4%)	
**Occupation**	Unemployed	37	8 (21.6%)	25 (67.6%)	4 (10.8%)	0.507
	Employed	162	25 (15.4%)	110 (67.9%)	27 (16.7%)	
**Educational level**	Low	5	2 (40%)	2 (40%)	1 (20%)	0.654
	Medium	60	9 (15%)	42 (70%)	9 (15%)	
	High	135	22 (16.3%)	92 (68.1%)	21 (15.6%)	
**MedDietScore**	Low adherence	54	7 (13%)	37 (68.5%)	10 (18.5%)	0.607
	High adherence	146	26 (17.8%)	99 (67.8%)	21 (14.4%)	
**Depressive symptomatology (BDI-II)**	Minimal depressive symptoms	156	26 (16.7%)	107 (68.6%)	23 (14.7%)	0.856
	Depressive symptoms	44	7 (15.9%)	29 (65.9%)	8 (18.2%)	
**Anxiety (STAI-6)**	Normal level	99	17 (17.2%)	64 (64.2%)	18 (18.2%)	0.525
	High level	10	16 (15.8%)	72 (71.3%)	13 (12.9%)	
**Child dietary counseling**	Yes	25	3 (12%)	17 (68%)	5 (20%)	0.696
	No	175	30 (17.1%)	119 (68%)	31 (15.5%)	

**Table 2:** Comparison of maternal parameters between children with and without anxiety.

Maternal Variables	N	Category/Mean Value	Anxiety (Median [IQR])	No Anxiety (Median [IQR])	*p*-Value
**Age**	200	38.02	38 [6.75]	38 [4]	0.213
**Place of residence**	Urban (Ν = 29)	1:Urban / 2:Rural	2 [0]	2 [0]	0.616
	Rural (Ν = 171)				
**Nationality**	Greek (Ν = 191)	1:Greek / 2:Other	1 [0]	1 [0]	0.248
	Other (Ν = 9)				
**Number of children**	200	2.03	2 [1]	2 [1]	0.356
**Weight (kg)**	200	67.56	65 [19.75]	64.75 [16]	0.771
**Height (cm)**	200	164.81	165 [10]	165 [8]	0.199
**ΒΜΙ**	200	24.87	23.98 [6.13]	23.77 [6.14]	0.857
**Waist circumference**	200	84.48	80.5 [17]	80 [19.75]	0.833
**Hip circumference**	200	102.53	100 [12.75]	100.5 [14.5]	0.865
**WHR**	200	4,95	0.82 [0.093]	0.80 [0.08]	0.462
**Pre-pregnancy weight**	200	62.55	60 [14.75]	58.5 [10]	0.286
**Weight before delivery**	200	75.36	74 [17.25]	72 [16.5]	0.181
**GWG**	200	12.81	13 [7.25]	10 [8.5]	0.074
**Exercise frequency/per week**	200	1.64	2 [3]	1 [2]	0.937
**Smoking status**	Yes (Ν = 41)	1:Yes / 2:No	2 [0]	2 [0]	0.333
	No (Ν = 159)				
**Illness**	Yes (Ν = 39)	1:Yes / 2:No	2 [0]	2 [0]	0.668
	No (Ν = 161)				
**Medication use**	Yes (Ν = 33)	1:Yes / 2:No	2 [0]	2 [0]	0.747
	No (Ν = 167)				
**Diet type**	Non-specific diet (Ν = 191)	1:Non-specific / 2: Special diet	1 [0]	1 [0]	0.261
	Special diet (Ν = 9)				
**Marital status**	Single (Ν = 5)	1: Single / 2: Married /3: Divorced	2 [0]	2 [0]	0.271
	Married (Ν = 188)				
	Divorced (Ν = 7)				
**Economic status**	Low (Ν = 82)	1:Low / 2:Medium / 3:High	2 [1]	2 [1]	0.430
	Medium (Ν = 105)				
	High (Ν = 13)				
**Employment status**	Unemployed (Ν = 37)	0: Unemployed / 1: Employed	1 [0]	1 [0]	0.328
	Employed (Ν = 162)				
**Educational level**	Low (Ν = 5)	1:Low / 2:Medium/3: High	3 [1]	3 [1]	0.921
	Medium (Ν = 60)				
	High (Ν = 135)				
**MedDietScore**	Low compliance (Ν = 54)	1: Low / 2: High compliance	2 [1]	2 [1]	0.505
	High compliance (Ν = 146)				
**Depressive symptomatology (BDI)**	Minimal symptoms (Ν = 156)	Minimal symptoms / 2: Depressive symptoms	1 [0]	1 [1]	<0.001
	Depressive symptoms (Ν = 44)				
**Anxiety (STAI-6)**	Normal (Ν = 99)	1:Normal / 2:High level	2 [0]	1 [1]	<0.001
	High level (Ν = 10)				
**Child dietary counseling**	Yes (Ν = 25)	1:Yes / 2:No	2 [0]	2 [0]	1
	No (Ν = 175)				

**Table 3:** Logistic regression estimates of maternal factors predicting child anxiety status.

Maternal Variables	B	Odds Ratio (OR)	95%CI	*p*-Value	N
**Constant**	−4.017			0.000	200
**Pre-pregnancy weight**	−0.376	0.687	0.392–1.202	0.189	200
**Depressive symptoms (BDI)**	0.998	2.712	1.222–6.020	0.014	200
**Anxiety (STAI-6)**	1.676	5.346	2.471–11.565	0.000	200
**Height**	0.225	1.253	0.705–2.226	0.442	200
**GWG**	–0.041	0.960	0.908–1.014	0.145	200

## Data Availability

All data is available upon request from the corresponding author.
